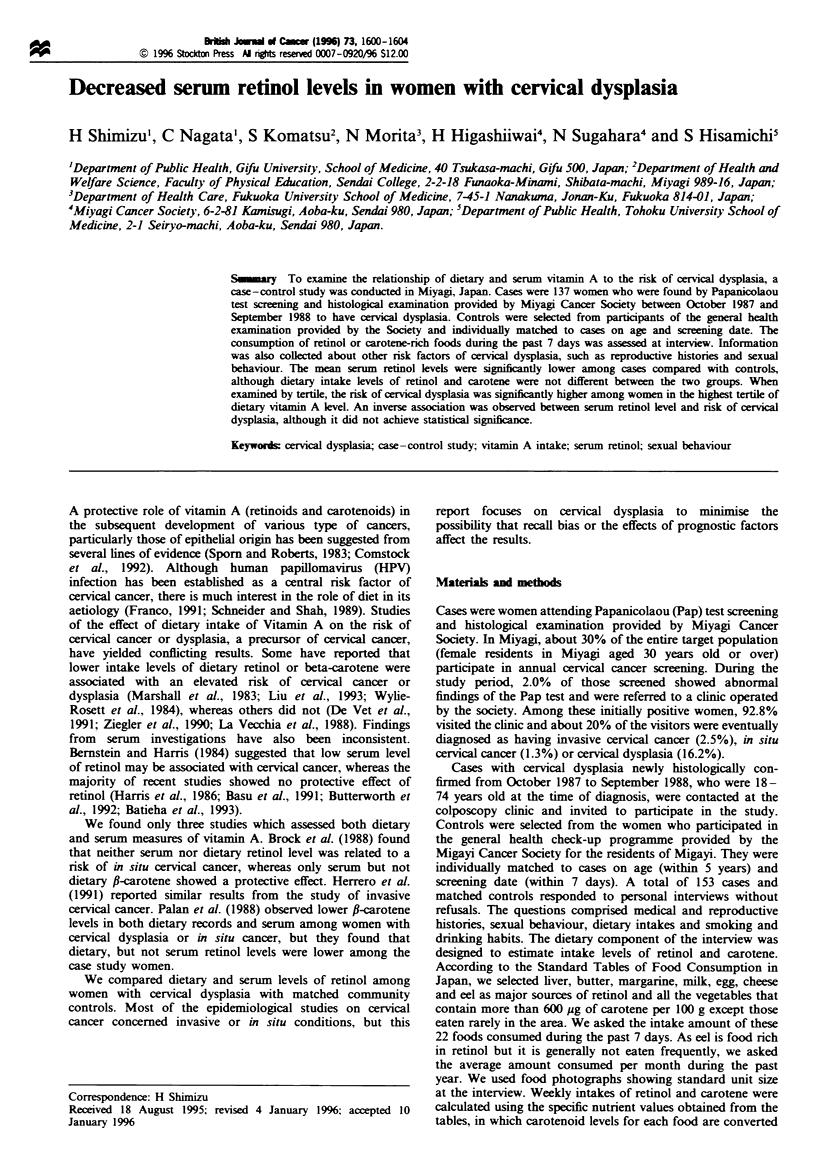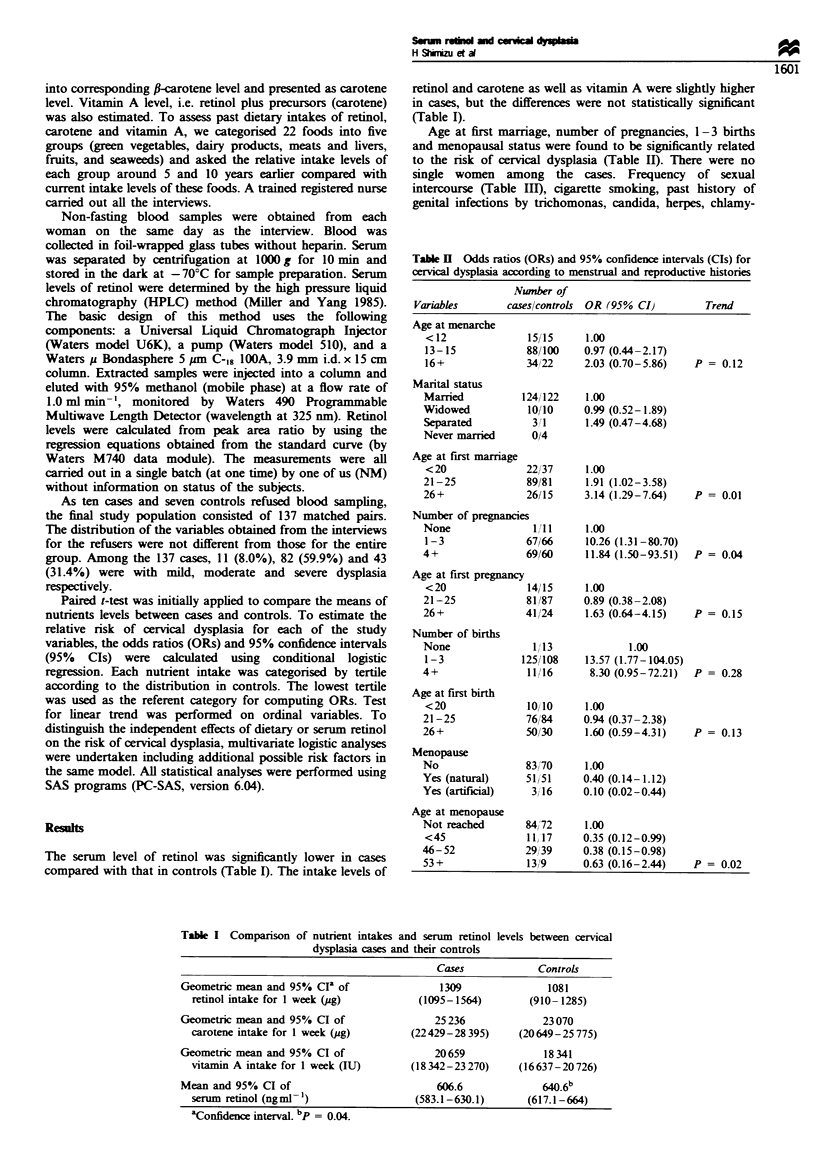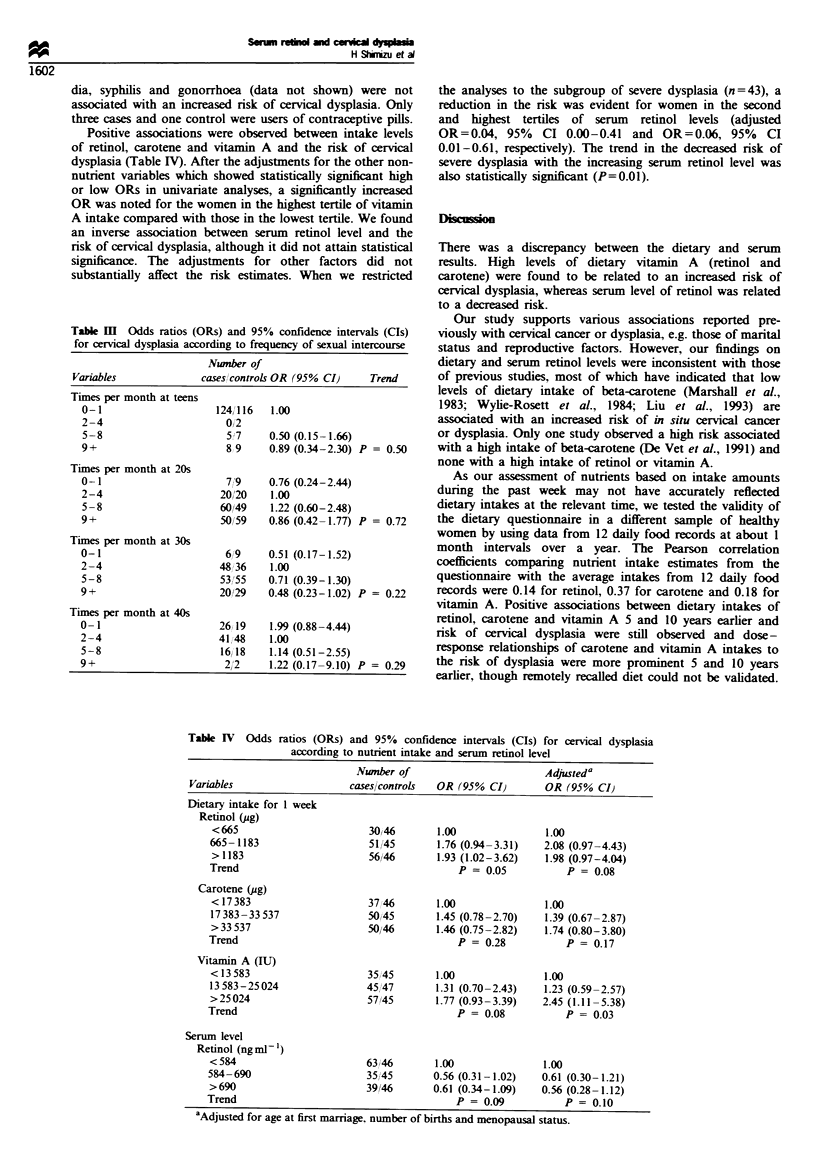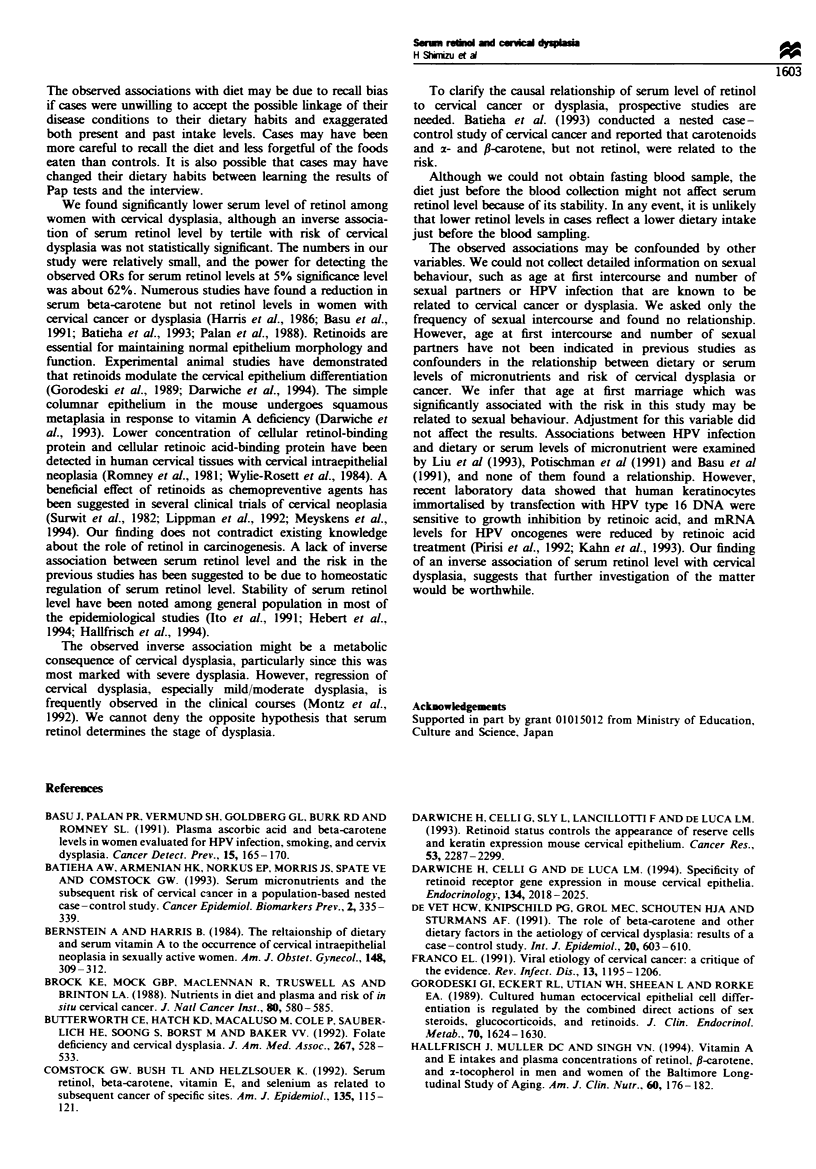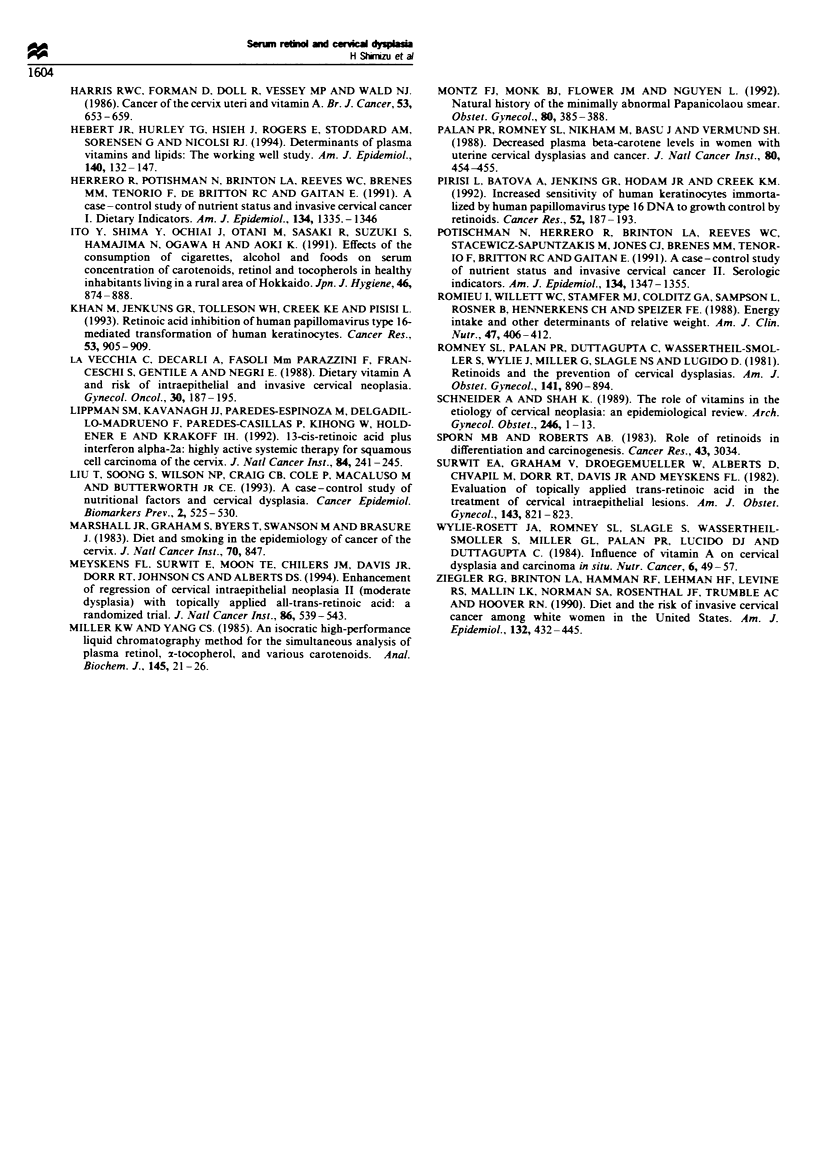# Decreased serum retinol levels in women with cervical dysplasia.

**DOI:** 10.1038/bjc.1996.301

**Published:** 1996-06

**Authors:** H. Shimizu, C. Nagata, S. Komatsu, N. Morita, H. Higashiiwai, N. Sugahara, S. Hisamichi

**Affiliations:** Department of Public Health, Gifu University, School of Medicine, Japan.

## Abstract

To examine the relationship of dietary and serum vitamin A to the risk of cervical dysplasia, a case-control study was conducted in Miyagi, Japan. Cases were 137 women who were found by Papanicolaou test screening and histological examination provided by Miyagi Cancer Society between October 1987 and September 1988 to have cervical dysplasia. Controls were selected from participants of the general health examination provided by the Society and individually matched to cases on age and screening date. The consumption of retinol or carotene-rich foods during the past 7 days was assessed at interview. Information was also collected about other risk factors of cervical dysplasia, such as reproductive histories and sexual behaviour. The mean serum retinol levels were significantly lower among cases compared with controls, although dietary intake levels of retinol and carotene were not different between the two groups. When examined by tertile, the risk of cervical dysplasia was significantly higher among women in the highest tertile of dietary vitamin A level. An inverse association was observed between serum retinol level and risk of cervical dysplasia, although it did not achieve statistical significance.


© 1996 Stockton Press All rights reserved 0007-0920/96 $12.00

### Decreased serum retinol levels in women with cervical dysplasia

H Shimizu[1], C Nagata[1], S Komatsu[2], N Morita[3], H Higashiiwai[4], N Sugahara[4] and S Hisamichi[5]

[1]*Department of Public Health, Gifu University, School of Medicine, 40 Tsukasa-machi, Gifu 500, Japan;* [2]*Department of Health and Welfare Science, Faculty of Physical Education, Sendai College, 2-2-18 Funaoka-Minami, Shibata-machi, Miyagi 989-16, Japan;* [3]*Department of Health Care, Fukuoka University School of Medicine, 7-45-1 Nanakuma, Jonan-Ku, Fukuoka 814-01, Japan;* [4]*Miyagi Cancer Society, 6-2-81 Kamisugi, Aoba-ku, Sendai 980, Japan;* [5]*Department of Public Health, Tohoku University School of Medicine, 2-1 Seiryo-machi, Aoba-ku, Sendai 980, Japan.*

**Summary** To examine the relationship of dietary and serum vitamin A to the risk of cervical dysplasia, a case-control study was conducted in Miyagi, Japan. Cases were 137 women who were found by Papanicolaou test screening and histological examination provided by Miyagi Cancer Society between October 1987 and September 1988 to have cervical dysplasia. Controls were selected from participants of the general health examination provided by the Society and individually matched to cases on age and screening date. The consumption of retinol or carotene-rich foods during the past 7 days was assessed at interview. Information was also collected about other risk factors of cervical dysplasia, such as reproductive histories and sexual behaviour. The mean serum retinol levels were significantly lower among cases compared with controls, although dietary intake levels of retinol and carotene were not different between the two groups. When examined by tertile, the risk of cervical dysplasia was significantly higher among women in the highest tertile of dietary vitamin A level. An inverse association was observed between serum retinol level and risk of cervical dysplasia, although it did not achieve statistical significance.

**Keywords:** cervical dysplasia; case-control study; vitamin A intake; serum retinol; sexual behaviour

A protective role of vitamin A (retinoids and carotenoids) in the subsequent development of various type of cancers, particularly those of epithelial origin has been suggested from several lines of evidence (Sporn and Roberts, 1983; Comstock *et al.*, 1992). Although human papillomavirus (HPV) infection has been established as a central risk factor of cervical cancer, there is much interest in the role of diet in its aetiology (Franco, 1991; Schneider and Shah, 1989). Studies of the effect of dietary intake of Vitamin A on the risk of cervical cancer or dysplasia, a precursor of cervical cancer, have yielded conflicting results. Some have reported that lower intake levels of dietary retinol or beta-carotene were associated with an elevated risk of cervical cancer or dysplasia (Marshall *et al.*, 1983; Liu *et al.*, 1993; Wylie-Rosett *et al.*, 1984), whereas others did not (De Vet *et al.*, 1991; Ziegler *et al.*, 1990; La Vecchia *et al.*, 1988). Findings from serum investigations have also been inconsistent. Bernstein and Harris (1984) suggested that low serum level of retinol may be associated with cervical cancer, whereas the majority of recent studies showed no protective effect of retinol (Harris *et al.*, 1986; Basu *et al.*, 1991; Butterworth *et al.*, 1992; Batieha *et al.*, 1993).

We found only three studies which assessed both dietary and serum measures of vitamin A. Brock *et al.* (1988) found that neither serum nor dietary retinol level was related to a risk of *in situ* cervical cancer, whereas only serum but not dietary $\beta$-carotene showed a protective effect. Herrero *et al.* (1991) reported similar results from the study of invasive cervical cancer. Palan *et al.* (1988) observed lower $\beta$-carotene levels in both dietary records and serum among women with cervical dysplasia or *in situ* cancer, but they found that dietary, but not serum retinol levels were lower among the case study women.

We compared dietary and serum levels of retinol among women with cervical dysplasia with matched community controls. Most of the epidemiological studies on cervical cancer concerned invasive or *in situ* conditions, but this report focuses on cervical dysplasia to minimise the possibility that recall bias or the effects of prognostic factors affect the results.

Correspondence: H Shimizu
Received 18 August 1995; revised 4 January 1996; accepted 10 January 1996

### Materials and methods

Cases were women attending Papanicolaou (Pap) test screening and histological examination provided by Miyagi Cancer Society. In Miyagi, about 30% of the entire target population (female residents in Miyagi aged 30 years old or over) participate in annual cervical cancer screening. During the study period, 2.0% of those screened showed abnormal findings of the Pap test and were referred to a clinic operated by the society. Among these initially positive women, 92.8% visited the clinic and about 20% of the visitors were eventually diagnosed as having invasive cervical cancer (2.5%), *in situ* cervical cancer (1.3%) or cervical dysplasia (16.2%).

Cases with cervical dysplasia newly histologically confirmed from October 1987 to September 1988, who were 18-74 years old at the time of diagnosis, were contacted at the colposcopy clinic and invited to participate in the study. Controls were selected from the women who participated in the general health check-up programme provided by the Migayi Cancer Society for the residents of Migayi. They were individually matched to cases on age (within 5 years) and screening date (within 7 days). A total of 153 cases and matched controls responded to personal interviews without refusals. The questions comprised medical and reproductive histories, sexual behaviour, dietary intakes and smoking and drinking habits. The dietary component of the interview was designed to estimate intake levels of retinol and carotene. According to the Standard Tables of Food Consumption in Japan, we selected liver, butter, margarine, milk, egg, cheese and eel as major sources of retinol and all the vegetables that contain more than 600 $\mu$g of carotene per 100 g except those eaten rarely in the area. We asked the intake amount of these 22 foods consumed during the past 7 days. As eel is food rich in retinol but it is generally not eaten frequently, we asked the average amount consumed per month during the past year. We used food photographs showing standard unit size at the interview. Weekly intakes of retinol and carotene were calculated using the specific nutrient values obtained from the tables, in which carotenoid levels for each food are converted into corresponding β-carotene level and presented as carotene level. Vitamin A level, i.e. retinol plus precursors (carotene) was also estimated. To assess past dietary intakes of retinol, carotene and vitamin A, we categorised 22 foods into five groups (green vegetables, dairy products, meats and livers, fruits, and seaweeds) and asked the relative intake levels of each group around 5 and 10 years earlier compared with current intake levels of these foods. A trained registered nurse carried out all the interviews.

Non-fasting blood samples were obtained from each woman on the same day as the interview. Blood was collected in foil-wrapped glass tubes without heparin. Serum was separated by centrifugation at 1000 *g* for 10 min and stored in the dark at −70°C for sample preparation. Serum levels of retinol were determined by the high pressure liquid chromatography (HPLC) method (Miller and Yang 1985). The basic design of this method uses the following components: a Universal Liquid Chromatograph Injector (Waters model U6K), a pump (Waters model 510), and a Waters μ Bondasphere 5 μm $C_{18}$ 100A, 3.9 mm i.d. x 15 cm column. Extracted samples were injected into a column and eluted with 95% methanol (mobile phase) at a flow rate of 1.0 ml $min^{-1}$, monitored by Waters 490 Programmable Multiwave Length Detector (wavelength at 325 nm). Retinol levels were calculated from peak area ratio by using the regression equations obtained from the standard curve (by Waters M740 data module). The measurements were all carried out in a single batch (at one time) by one of us (NM) without information on status of the subjects.

As ten cases and seven controls refused blood sampling, the final study population consisted of 137 matched pairs. The distribution of the variables obtained from the interviews for the refusers were not different from those for the entire group. Among the 137 cases, 11 (8.0%), 82 (59.9%) and 43 (31.4%) were with mild, moderate and severe dysplasia respectively.

Paired *t*-test was initially applied to compare the means of nutrients levels between cases and controls. To estimate the relative risk of cervical dysplasia for each of the study variables, the odds ratios (ORs) and 95% confidence intervals (95% CIs) were calculated using conditional logistic regression. Each nutrient intake was categorised by tertile according to the distribution in controls. The lowest tertile was used as the referent category for computing ORs. Test for linear trend was performed on ordinal variables. To distinguish the independent effects of dietary or serum retinol on the risk of cervical dysplasia, multivariate logistic analyses were undertaken including additional possible risk factors in the same model. All statistical analyses were performed using SAS programs (PC-SAS, version 6.04).

### Results

The serum level of retinol was significantly lower in cases compared with that in controls (Table I). The intake levels of retinol and carotene as well as vitamin A were slightly higher in cases, but the differences were not statistically significant (Table I).

Age at first marriage, number of pregnancies, 1−3 births and menopausal status were found to be significantly related to the risk of cervical dysplasia (Table II). There were no single women among the cases. Frequency of sexual intercourse (Table III), cigarette smoking, past history of genital infections by trichomonas, candida, herpes, chlamy-

**Table II** Odds ratios (ORs) and 95% confidence intervals (CIs) for cervical dysplasia according to menstrual and reproductive histories

| *Variables* | *Number of cases/controls* | *OR (95% CI)* | *Trend* |
|---|---|---|---|
| Age at menarche | | | |
| <12 | 15/15 | 1.00 | |
| 13−15 | 88/100 | 0.97 (0.44−2.17) | |
| 16+ | 34/22 | 2.03 (0.70−5.86) | $P$ = 0.12 |
| Marital status | | | |
| Married | 124/122 | 1.00 | |
| Widowed | 10/10 | 0.99 (0.52−1.89) | |
| Separated | 3/1 | 1.49 (0.47−4.68) | |
| Never married | 0/4 | | |
| Age at first marriage | | | |
| <20 | 22/37 | 1.00 | |
| 21−25 | 89/81 | 1.91 (1.02−3.58) | |
| 26+ | 26/15 | 3.14 (1.29−7.64) | $P$ = 0.01 |
| Number of pregnancies | | | |
| None | 1/11 | 1.00 | |
| 1−3 | 67/66 | 10.26 (1.31−80.70) | |
| 4+ | 69/60 | 11.84 (1.50−93.51) | $P$ = 0.04 |
| Age at first pregnancy | | | |
| <20 | 14/15 | 1.00 | |
| 21−25 | 81/87 | 0.89 (0.38−2.08) | |
| 26+ | 41/24 | 1.63 (0.64−4.15) | $P$ = 0.15 |
| Number of births | | | |
| None | 1/13 | 1.00 | |
| 1−3 | 125/108 | 13.57 (1.77−104.05) | |
| 4+ | 11/16 | 8.30 (0.95−72.21) | $P$ = 0.28 |
| Age at first birth | | | |
| <20 | 10/10 | 1.00 | |
| 21−25 | 76/84 | 0.94 (0.37−2.38) | |
| 26+ | 50/30 | 1.60 (0.59−4.31) | $P$ = 0.13 |
| Menopause | | | |
| No | 83/70 | 1.00 | |
| Yes (natural) | 51/51 | 0.40 (0.14−1.12) | |
| Yes (artificial) | 3/16 | 0.10 (0.02−0.44) | |
| Age at menopause | | | |
| Not reached | 84/72 | 1.00 | |
| <45 | 11/17 | 0.35 (0.12−0.99) | |
| 46−52 | 29/39 | 0.38 (0.15−0.98) | |
| 53+ | 13/9 | 0.63 (0.16−2.44) | $P$ = 0.02 |

**Table I** Comparison of nutrient intakes and serum retinol levels between cervical dysplasia cases and their controls

| | *Cases* | *Controls* |
|---|---|---|
| Geometric mean and 95% CI[a] of retinol intake for 1 week (μg) | 1309 (1095−1564) | 1081 (910−1285) |
| Geometric mean and 95% CI of carotene intake for 1 week (μg) | 25 236 (22 429−28 395) | 23 070 (20 649−25 775) |
| Geometric mean and 95% CI of vitamin A intake for 1 week (IU) | 20 659 (18 342−23 270) | 18 341 (16 637−20 726) |
| Mean and 95% CI of serum retinol (ng $ml^{-1}$) | 606.6 (583.1−630.1) | 640.6[b] (617.1−664) |

[a]Confidence interval. [b]$P$ = 0.04.

dia, syphilis and gonorrhoea (data not shown) were not associated with an increased risk of cervical dysplasia. Only three cases and one control were users of contraceptive pills.

Positive associations were observed between intake levels of retinol, carotene and vitamin A and the risk of cervical dysplasia (Table IV). After the adjustments for the other non-nutrient variables which showed statistically significant high or low ORs in univariate analyses, a significantly increased OR was noted for the women in the highest tertile of vitamin A intake compared with those in the lowest tertile. We found an inverse association between serum retinol level and the risk of cervical dysplasia, although it did not attain statistical significance. The adjustments for other factors did not substantially affect the risk estimates. When we restricted the analyses to the subgroup of severe dysplasia ($n=43$), a reduction in the risk was evident for women in the second and highest tertiles of serum retinol levels (adjusted OR=0.04, 95% CI 0.00–0.41 and OR=0.06, 95% CI 0.01–0.61, respectively). The trend in the decreased risk of severe dysplasia with the increasing serum retinol level was also statistically significant ($P=0.01$).

**Table III** Odds ratios (ORs) and 95% confidence intervals (CIs) for cervical dysplasia according to frequency of sexual intercourse

| *Variables* | *Number of cases/controls* | *OR (95% CI)* | *Trend* |
|---|---|---|---|
| Times per month at teens | | | |
| 0–1 | 124/116 | 1.00 | |
| 2–4 | 0/2 | | |
| 5–8 | 5/7 | 0.50 (0.15–1.66) | |
| 9+ | 8/9 | 0.89 (0.34–2.30) | P = 0.50 |
| Times per month at 20s | | | |
| 0–1 | 7/9 | 0.76 (0.24–2.44) | |
| 2–4 | 20/20 | 1.00 | |
| 5–8 | 60/49 | 1.22 (0.60–2.48) | |
| 9+ | 50/59 | 0.86 (0.42–1.77) | P = 0.72 |
| Times per month at 30s | | | |
| 0–1 | 6/9 | 0.51 (0.17–1.52) | |
| 2–4 | 48/36 | 1.00 | |
| 5–8 | 53/55 | 0.71 (0.39–1.30) | |
| 9+ | 20/29 | 0.48 (0.23–1.02) | P = 0.22 |
| Times per month at 40s | | | |
| 0–1 | 26/19 | 1.99 (0.88–4.44) | |
| 2–4 | 41/48 | 1.00 | |
| 5–8 | 16/18 | 1.14 (0.51–2.55) | |
| 9+ | 2/2 | 1.22 (0.17–9.10) | P = 0.29 |

### Discussion

There was a discrepancy between the dietary and serum results. High levels of dietary vitamin A (retinol and carotene) were found to be related to an increased risk of cervical dysplasia, whereas serum level of retinol was related to a decreased risk.

Our study supports various associations reported previously with cervical cancer or dysplasia, e.g. those of marital status and reproductive factors. However, our findings on dietary and serum retinol levels were inconsistent with those of previous studies, most of which have indicated that low levels of dietary intake of beta-carotene (Marshall *et al.*, 1983; Wylie-Rosett *et al.*, 1984; Liu *et al.*, 1993) are associated with an increased risk of *in situ* cervical cancer or dysplasia. Only one study observed a high risk associated with a high intake of beta-carotene (De Vet *et al.*, 1991) and none with a high intake of retinol or vitamin A.

As our assessment of nutrients based on intake amounts during the past week may not have accurately reflected dietary intakes at the relevant time, we tested the validity of the dietary questionnaire in a different sample of healthy women by using data from 12 daily food records at about 1 month intervals over a year. The Pearson correlation coefficients comparing nutrient intake estimates from the questionnaire with the average intakes from 12 daily food records were 0.14 for retinol, 0.37 for carotene and 0.18 for vitamin A. Positive associations between dietary intakes of retinol, carotene and vitamin A 5 and 10 years earlier and risk of cervical dysplasia were still observed and dose–response relationships of carotene and vitamin A intakes to the risk of dysplasia were more prominent 5 and 10 years earlier, though remotely recalled diet could not be validated.

**Table IV** Odds ratios (ORs) and 95% confidence intervals (CIs) for cervical dysplasia according to nutrient intake and serum retinol level

| *Variables* | *Number of cases/controls* | *OR (95% CI)* | *Adjusted[a] OR (95% CI)* |
|---|---|---|---|
| Dietary intake for 1 week | | | |
| Retinol ($\mu$g) | | | |
| <665 | 30/46 | 1.00 | 1.00 |
| 665–1183 | 51/45 | 1.76 (0.94–3.31) | 2.08 (0.97–4.43) |
| >1183 | 56/46 | 1.93 (1.02–3.62) | 1.98 (0.97–4.04) |
| Trend | | P = 0.05 | P = 0.08 |
| Carotene ($\mu$g) | | | |
| <17383 | 37/46 | 1.00 | 1.00 |
| 17383–33537 | 50/45 | 1.45 (0.78–2.70) | 1.39 (0.67–2.87) |
| >33537 | 50/46 | 1.46 (0.75–2.82) | 1.74 (0.80–3.80) |
| Trend | | P = 0.28 | P = 0.17 |
| Vitamin A (IU) | | | |
| <13583 | 35/45 | 1.00 | 1.00 |
| 13583–25024 | 45/47 | 1.31 (0.70–2.43) | 1.23 (0.59–2.57) |
| >25024 | 57/45 | 1.77 (0.93–3.39) | 2.45 (1.11–5.38) |
| Trend | | P = 0.08 | P = 0.03 |
| Serum level | | | |
| Retinol (ng ml$^{-1}$) | | | |
| <584 | 63/46 | 1.00 | 1.00 |
| 584–690 | 35/45 | 0.56 (0.31–1.02) | 0.61 (0.30–1.21) |
| >690 | 39/46 | 0.61 (0.34–1.09) | 0.56 (0.28–1.12) |
| Trend | | P = 0.09 | P = 0.10 |

[a]Adjusted for age at first marriage, number of births and menopausal status.

The observed associations with diet may be due to recall bias if cases were unwilling to accept the possible linkage of their disease conditions to their dietary habits and exaggerated both present and past intake levels. Cases may have been more careful to recall the diet and less forgetful of the foods eaten than controls. It is also possible that cases may have changed their dietary habits between learning the results of Pap tests and the interview.

We found significantly lower serum level of retinol among women with cervical dysplasia, although an inverse association of serum retinol level by tertile with risk of cervical dysplasia was not statistically significant. The numbers in our study were relatively small, and the power for detecting the observed ORs for serum retinol levels at 5% significance level was about 62%. Numerous studies have found a reduction in serum beta-carotene but not retinol levels in women with cervical cancer or dysplasia (Harris *et al.*, 1986; Basu *et al.*, 1991; Batieha *et al.*, 1993; Palan *et al.*, 1988). Retinoids are essential for maintaining normal epithelium morphology and function. Experimental animal studies have demonstrated that retinoids modulate the cervical epithelium differentiation (Gorodeski *et al.*, 1989; Darwiche *et al.*, 1994). The simple columnar epithelium in the mouse undergoes squamous metaplasia in response to vitamin A deficiency (Darwiche *et al.*, 1993). Lower concentration of cellular retinol-binding protein and cellular retinoic acid-binding protein have been detected in human cervical tissues with cervical intraepithelial neoplasia (Romney *et al.*, 1981; Wylie-Rosett *et al.*, 1984). A beneficial effect of retinoids as chemopreventive agents has been suggested in several clinical trials of cervical neoplasia (Surwit *et al.*, 1982; Lippman *et al.*, 1992; Meyskens *et al.*, 1994). Our finding does not contradict existing knowledge about the role of retinol in carcinogenesis. A lack of inverse association between serum retinol level and the risk in the previous studies has been suggested to be due to homeostatic regulation of serum retinol level. Stability of serum retinol level have been noted among general population in most of the epidemiological studies (Ito *et al.*, 1991; Hebert *et al.*, 1994; Hallfrisch *et al.*, 1994).

The observed inverse association might be a metabolic consequence of cervical dysplasia, particularly since this was most marked with severe dysplasia. However, regression of cervical dysplasia, especially mild/moderate dysplasia, is frequently observed in the clinical courses (Montz *et al.*, 1992). We cannot deny the opposite hypothesis that serum retinol determines the stage of dysplasia.

To clarify the causal relationship of serum level of retinol to cervical cancer or dysplasia, prospective studies are needed. Batieha *et al.* (1993) conducted a nested case–control study of cervical cancer and reported that carotenoids and $\alpha$- and $\beta$-carotene, but not retinol, were related to the risk.

Although we could not obtain fasting blood sample, the diet just before the blood collection might not affect serum retinol level because of its stability. In any event, it is unlikely that lower retinol levels in cases reflect a lower dietary intake just before the blood sampling.

The observed associations may be confounded by other variables. We could not collect detailed information on sexual behaviour, such as age at first intercourse and number of sexual partners or HPV infection that are known to be related to cervical cancer or dysplasia. We asked only the frequency of sexual intercourse and found no relationship. However, age at first intercourse and number of sexual partners have not been indicated in previous studies as confounders in the relationship between dietary or serum levels of micronutrients and risk of cervical dysplasia or cancer. We infer that age at first marriage which was significantly associated with the risk in this study may be related to sexual behaviour. Adjustment for this variable did not affect the results. Associations between HPV infection and dietary or serum levels of micronutrient were examined by Liu *et al* (1993), Potischman *et al* (1991) and Basu *et al* (1991), and none of them found a relationship. However, recent laboratory data showed that human keratinocytes immortalised by transfection with HPV type 16 DNA were sensitive to growth inhibition by retinoic acid, and mRNA levels for HPV oncogenes were reduced by retinoic acid treatment (Pirisi *et al.*, 1992; Kahn *et al.*, 1993). Our finding of an inverse association of serum retinol level with cervical dysplasia, suggests that further investigation of the matter would be worthwhile.

**Acknowledgements**

Supported in part by grant 01015012 from Ministry of Education, Culture and Science, Japan

### References

BASU J, PALAN PR, VERMUND SH, GOLDBERG GL, BURK RD AND ROMNEY SL. (1991). Plasma ascorbic acid and beta-carotene levels in women evaluated for HPV infection, smoking, and cervix dysplasia. *Cancer Detect. Prev.*, **15**, 165–170.

BATIEHA AW, ARMENIAN HK, NORKUS EP, MORRIS JS, SPATE VE AND COMSTOCK GW. (1993). Serum micronutrients and the subsequent risk of cervical cancer in a population-based nested case–control study. *Cancer Epidemiol. Biomarkers Prev.*, **2**, 335–339.

BERNSTEIN A AND HARRIS B. (1984). The reltaionship of dietary and serum vitamin A to the occurrence of cervical intraepithelial neoplasia in sexually active women. *Am. J. Obstet. Gynecol.*, **148**, 309–312.

BROCK KE, MOCK GBP, MACLENNAN R, TRUSWELL AS AND BRINTON LA. (1988). Nutrients in diet and plasma and risk of *in situ* cervical cancer. *J. Natl Cancer Inst.*, **80**, 580–585.

BUTTERWORTH CE, HATCH KD, MACALUSO M, COLE P, SAUBERLICH HE, SOONG S, BORST M AND BAKER VV. (1992). Folate deficiency and cervical dysplasia. *J. Am. Med. Assoc.*, **267**, 528–533.

COMSTOCK GW, BUSH TL AND HELZLSOUER K. (1992). Serum retinol, beta-carotene, vitamin E, and selenium as related to subsequent cancer of specific sites. *Am. J. Epidemiol.*, **135**, 115–121.

DARWICHE H, CELLI G, SLY L, LANCILLOTTI F AND DE LUCA LM. (1993). Retinoid status controls the appearance of reserve cells and keratin expression mouse cervical epithelium. *Cancer Res.*, **53**, 2287–2299.

DARWICHE H, CELLI G AND DE LUCA LM. (1994). Specificity of retinoid receptor gene expression in mouse cervical epithelia. *Endocrinology*, **134**, 2018–2025.

DE VET HCW, KNIPSCHILD PG, GROL MEC, SCHOUTEN HJA AND STURMANS AF. (1991). The role of beta-carotene and other dietary factors in the aetiology of cervical dysplasia: results of a case–control study. *Int. J. Epidemiol.*, **20**, 603–610.

FRANCO EL. (1991). Viral etiology of cervical cancer: a critique of the evidence. *Rev. Infect. Dis.*, **13**, 1195–1206.

GORODESKI GI, ECKERT RL, UTIAN WH, SHEEAN L AND RORKE EA. (1989). Cultured human ectocervical epithelial cell differentiation is regulated by the combined direct actions of sex steroids, glucocorticoids, and retinoids. *J. Clin. Endocrinol. Metab.*, **70**, 1624–1630.

HALLFRISCH J, MULLER DC AND SINGH VN. (1994). Vitamin A and E intakes and plasma concentrations of retinol, $\beta$-carotene, and $\alpha$-tocopherol in men and women of the Baltimore Longtudinal Study of Aging. *Am. J. Clin. Nutr.*, **60**, 176–182.

HARRIS RWC, FORMAN D, DOLL R, VESSEY MP AND WALD NJ. (1986). Cancer of the cervix uteri and vitamin A. *Br. J. Cancer*, **53**, 653 – 659.

HEBERT JR, HURLEY TG, HSIEH J, ROGERS E, STODDARD AM, SORENSEN G AND NICOLSI RJ. (1994). Determinants of plasma vitamins and lipids: The working well study. *Am. J. Epidemiol.*, **140**, 132 – 147.

HERRERO R, POTISHMAN N, BRINTON LA, REEVES WC, BRENES MM, TENORIO F, DE BRITTON RC AND GAITAN E. (1991). A case – control study of nutrient status and invasive cervical cancer I. Dietary Indicators. *Am. J. Epidemiol.*, **134**, 1335. – 1346

ITO Y, SHIMA Y, OCHIAI J, OTANI M, SASAKI R, SUZUKI S, HAMAJIMA N, OGAWA H AND AOKI K. (1991). Effects of the consumption of cigarettes, alcohol and foods on serum concentration of carotenoids, retinol and tocopherols in healthy inhabitants living in a rural area of Hokkaido. *Jpn. J. Hygiene*, **46**, 874 – 888.

KHAN M, JENKUNS GR, TOLLESON WH, CREEK KE AND PISISI L. (1993). Retinoic acid inhibition of human papillomavirus type 16-mediated transformation of human keratinocytes. *Cancer Res.*, **53**, 905 – 909.

LA VECCHIA C, DECARLI A, FASOLI Mm PARAZZINI F, FRANCESCHI S, GENTILE A AND NEGRI E. (1988). Dietary vitamin A and risk of intraepithelial and invasive cervical neoplasia. *Gynecol. Oncol.*, **30**, 187 – 195.

LIPPMAN SM, KAVANAGH JJ, PAREDES-ESPINOZA M, DELGADILLO-MADRUENO F, PAREDES-CASILLAS P, KIHONG W, HOLDENER E AND KRAKOFF IH. (1992). 13-cis-retinoic acid plus interferon alpha-2a: highly active systemic therapy for squamous cell carcinoma of the cervix. *J. Natl Cancer Inst.*, **84**, 241 – 245.

LIU T, SOONG S, WILSON NP, CRAIG CB, COLE P, MACALUSO M AND BUTTERWORTH JR CE. (1993). A case – control study of nutritional factors and cervical dysplasia. *Cancer Epidemiol. Biomarkers Prev.*, **2**, 525 – 530.

MARSHALL JR, GRAHAM S, BYERS T, SWANSON M AND BRASURE J. (1983). Diet and smoking in the epidemiology of cancer of the cervix. *J. Natl Cancer Inst.*, **70**, 847.

MEYSKENS FL, SURWIT E, MOON TE, CHILERS JM, DAVIS JR, DORR RT, JOHNSON CS AND ALBERTS DS. (1994). Enhancement of regression of cervical intraepithelial neoplasia II (moderate dysplasia) with topically applied all-trans-retinoic acid: a randomized trial. *J. Natl Cancer Inst.*, **86**, 539 – 543.

MILLER KW AND YANG CS. (1985). An isocratic high-performance liquid chromatography method for the simultaneous analysis of plasma retinol, α-tocopherol, and various carotenoids. *Anal. Biochem. J.*, **145**, 21 – 26.

MONTZ FJ, MONK BJ, FLOWER JM AND NGUYEN L. (1992). Natural history of the minimally abnormal Papanicolaou smear. *Obstet. Gynecol.*, **80**, 385 – 388.

PALAN PR, ROMNEY SL, NIKHAM M, BASU J AND VERMUND SH. (1988). Decreased plasma beta-carotene levels in women with uterine cervical dysplasias and cancer. *J. Natl Cancer Inst.*, **80**, 454 – 455.

PIRISI L, BATOVA A, JENKINS GR, HODAM JR AND CREEK KM. (1992). Increased sensitivity of human keratinocytes immortalized by human papillomavirus type 16 DNA to growth control by retinoids. *Cancer Res.*, **52**, 187 – 193.

POTISCHMAN N, HERRERO R, BRINTON LA, REEVES WC, STACEWICZ-SAPUNTZAKIS M, JONES CJ, BRENES MM, TENORIO F, BRITTON RC AND GAITAN E. (1991). A case – control study of nutrient status and invasive cervical cancer II. Serologic indicators. *Am. J. Epidemiol.*, **134**, 1347 – 1355.

ROMIEU I, WILLETT WC, STAMFER MJ, COLDITZ GA, SAMPSON L, ROSNER B, HENNERKENS CH AND SPEIZER FE. (1988). Energy intake and other determinants of relative weight. *Am. J. Clin. Nutr.*, **47**, 406 – 412.

ROMNEY SL, PALAN PR, DUTTAGUPTA C, WASSERTHEIL-SMOLLER S, WYLIE J, MILLER G, SLAGLE NS AND LUGIDO D. (1981). Retinoids and the prevention of cervical dysplasias. *Am. J. Obstet. Gynecol.*, **141**, 890 – 894.

SCHNEIDER A AND SHAH K. (1989). The role of vitamins in the etiology of cervical neoplasia: an epidemiological review. *Arch. Gynecol. Obstet.*, **246**, 1 – 13.

SPORN MB AND ROBERTS AB. (1983). Role of retinoids in differentiation and carcinogenesis. *Cancer Res.*, **43**, 3034.

SURWIT EA, GRAHAM V, DROEGEMUELLER W, ALBERTS D, CHVAPIL M, DORR RT, DAVIS JR AND MEYSKENS FL. (1982). Evaluation of topically applied trans-retinoic acid in the treatment of cervical intraepithelial lesions. *Am. J. Obstet. Gynecol.*, **143**, 821 – 823.

WYLIE-ROSETT JA, ROMNEY SL, SLAGLE S, WASSERTHEIL-SMOLLER S, MILLER GL, PALAN PR, LUCIDO DJ AND DUTTAGUPTA C. (1984). Influence of vitamin A on cervical dysplasia and carcinoma *in situ*. *Nutr. Cancer*, **6**, 49 – 57.

ZIEGLER RG, BRINTON LA, HAMMAN RF, LEHMAN HF, LEVINE RS, MALLIN LK, NORMAN SA, ROSENTHAL JF, TRUMBLE AC AND HOOVER RN. (1990). Diet and the risk of invasive cervical cancer among white women in the United States. *Am. J. Epidemiol.*, **132**, 432 – 445.